# Leveraging a collaborative consortium model of mentee/mentor training to foster career progression of underrepresented postdoctoral researchers and promote institutional diversity and inclusion

**DOI:** 10.1371/journal.pone.0238518

**Published:** 2020-09-01

**Authors:** Laurie E. Risner, Xenia K. Morin, Evelyn S. Erenrich, Philip S. Clifford, Jeffrey Franke, Imogen Hurley, Nancy B. Schwartz

**Affiliations:** 1 Department of Pediatrics, University of Chicago, Chicago, Illinois, United States of America; 2 Department of Plant Biology, Rutgers University–New Brunswick, New Brunswick, New Jersey, United States of America; 3 School of Graduate Studies, Rutgers University–New Brunswick, New Brunswick, New Jersey, United States of America; 4 College of Applied Health Sciences, University of Illinois at Chicago, Chicago, Illinois, United States of America; 5 College of Behavioral and Social Sciences, University of Maryland, College Park, Maryland, United States of America; 6 Office of Postdoctoral Studies, University of Wisconsin-Madison, Madison, Wisconsin, United States of America; 7 Department of Pediatrics and Department of Biochemistry and Molecular Biology, University of Chicago, Chicago, Illinois, United States of America; Medical College of Wisconsin - Central Wisconsin Campus, UNITED STATES

## Abstract

Changing institutional culture to be more diverse and inclusive within the biomedical academic community is difficult for many reasons. Herein we present evidence that a collaborative model involving multiple institutions of higher education can initiate and execute individual institutional change directed at enhancing diversity and inclusion at the postdoctoral researcher (postdoc) and junior faculty level by implementing evidence-based mentoring practices. A higher education consortium, the Big Ten Academic Alliance, invited individual member institutions to send participants to one of two types of annual mentor training: 1) “Mentoring-Up” training for postdocs, a majority of whom were from underrepresented groups; 2) Mentor Facilitator training—a train-the-trainer model—for faculty and senior leadership. From 2016 to 2019, 102 postdocs and 160 senior faculty and administrative leaders participated. Postdocs reported improvements in their mentoring proficiency (87%) and improved relationships with their PIs (71%). 29% of postdoc respondents transitioned to faculty positions, and 85% of these were underrepresented and 75% were female. 59 out of the 120 faculty and administrators (49%) trained in the first three years provided mentor training on their campuses to over 3000 undergraduate and graduate students, postdocs and faculty within the project period. We conclude that early stage biomedical professionals as well as individual institutions of higher education benefited significantly from this collaborative mentee/mentor training model

## Introduction

It has long been recognized that diversifying the biomedical workforce is essential for advancing the research enterprise, but how to go about this transformation has been a challenge for many institutions of higher education. While the numbers of individuals from underrepresented (UR) groups (women, traditionally underrepresented minorities, and persons with disabilities) earning STEM doctorates has increased, the proportion of their employment in senior or leadership positions in private or academic settings has not changed significantly over the past 20 years [1, 2], suggesting that just increasing UR representation in the pipeline is insufficient [3–5]. In order to understand the paucity of diversity, especially in academia, some studies have focused on social/cognitive barriers to career progression for UR trainees [6–12], with interventions directed to the individual to address many of these barriers [13]. However, one area of postdoc development that has only recently been addressed is to optimize postdocs’ dual mentee and mentor roles via “Mentoring-Up” training [14]. Each module of this curriculum is considered from the perspective of the postdoc as a current mentee attempting to get the most out of their relationship with their PI/Mentor, and then flips to consideration of the concepts as a future and/or current mentor. The goal is to empower postdocs to be active participants in their mentoring relationships by emphasizing the mentees’ contributions in shaping more productive interactions that then can be built upon to develop their own skills as a future mentor. A more recent study reported a positive link between students’ skill development trajectory and the active engagement of postdoctoral researchers in lab discussions, although postdocs typically have no formal mentor role [15] and little formal mentorship training [16]. Thus, further examination of the impact of exposure to mentor competencies on postdocs’ personal and professional career development via the validated but under-utilized “Mentoring-Up” training program is warranted.

As well, multiple institutional factors contributing to the lack of diversity in higher education have been investigated/analyzed [17–23]. It is increasingly being recognized that “diversity initiatives without inclusivity are not sufficient” [24]. Inclusivity, which in turn enhances trainees’ self-efficacy, involves changing the environment in which trainees are embedded by creating equitable scientific communities in which all scientists feel welcomed and valued. Toward that end, a major initiative to improving institutional culture is the provision of research mentor training, which has been shown to: i.) improve the success of researchers-in-training at all levels [25]; ii.) lead to academic persistence [26]; and iii.) improve retention in academia [27]. More recent studies have shown effective mentorship is increasingly correlated with enhanced mentee productivity, persistence, self-efficacy, career satisfaction and success [15, 16]. As a result of these demonstrated positive outcomes, research mentor training, with a focus on mentor facilitator training at the faculty level to promote dissemination and implementation broadly, has increased [28–30].

Even though the benefits of better mentoring to career success of graduate students, postdocs and junior faculty is important, and there is ample evidence that changes can be made to improve the mentoring environment, implementation has been slow. This is especially unfortunate for postdocs, as it has been documented that: i.) many women leave STEM careers during the critical postdoc career stage [31]); ii.) current training environments do not provide adequate mentor support, especially for UR trainees [12]; iii.) improved mentoring for faculty throughout their careers is increasingly being recognized as essential for faculty productivity and success as well [32]; iv.) changes to NIH procedures now require a commitment of effective mentoring associated with NIH training programs (https://grants.nih.gov/grants/guide/pa-files/PAR-17-341.html); and v.) superb evidence-based *Entering Mentoring* curricula have been available for over a decade [33], with new models being developed, including one specifically focused on diverse populations [34]. Nevertheless, there appear to be substantial barriers to implementing mentor training, both conceptual (lack of knowledge, experience, or confidence) and tangible (lack of facilitators, staff assistance, financial resources, incentives/rewards, institutional support, time, and low numbers of UR trainees or committed faculty at any one institution, etc.). These barriers impede implementation and dissemination of mentor training, especially for UR trainees [29].

To address many of these barriers, we have taken a collaborative, multi-institutional approach by leveraging the National Research Mentoring Network (NRMN), the Big Ten Academic Alliance (formerly the Committee on Institutional Cooperation (CIC)) Academic Network (CAN), an NIH supplement and NSF Alliance for Graduate Education and the Professoriate (AGEP) support. NRMN-CAN workshops brought together groups annually to provide 1) Mentor Facilitator training for faculty and senior administrators, and 2) “Mentoring-Up” training for postdocs—the Mentors of the future. This paper reports on the implementation of the NRMN-CAN model, as well as the metrics by which we assessed the success of this multi-year pilot study. We conclude that collaboration across an academic network that allows a critical mass of UR postdocs as well as committed faculty and senior staff to participate in mentor training venues, ultimately will lead to meaningful institutional change.

## Methods

### NRMN-CAN approach

The National Research Mentoring Network (NRMN)–CIC Academic Network (CAN) initiative was funded by a supplement from the National Research Mentoring Network (U54GM119023; 5101965–6), (https://nrmnet.net/), in addition to some postdoc travel support from the National Science Foundation under an AGEP-Transformation award #1309028. This study was granted exempt status from the Institutional Review Board at the University of Chicago under IRB Protocol Number 15–1724. Consent was not obtained as data were analyzed anonymously. NRMN-CAN addressed the need for greater diversity in the biomedical academic research workforce by: 1) providing professional development, grant-writing, and mentor training to predominantly UR Early Stage Investigators (postdocs and junior faculty); 2) training cohorts of faculty grantsmanship coaches and establishing campus-based grant-writing groups; and 3) directly addressing the benefits and challenges of diversity, inclusivity, and culture within mentoring relationships by training faculty and senior administrators as facilitators for a curriculum of best practices in mentoring. This paper reports the results of our four-year experience using this multi-institution model for intensive face-to-face mentor training of postdocs, as well as mentor facilitator training of senior colleagues at the Big Ten Conference Center near Chicago, with follow-up implementation of mentor-training sessions on home campuses to foster capacity-building, dissemination, and sustainability of good mentoring practices.

### Registration

For both types of Mentor Training, postdoc, faculty and senior administrator applicants registered and created a personal profile on the NRMNet.net website, which collected data about demographics, previous training and research background, and current position. In addition, the applicants completed an online registration form specific to the NRMN-CAN training workshop to indicate their motivation, expectations for participation in the training options and future plans for using what they learned. Outcomes tracking was conducted by the NRMN-CAN Administrative Core.

### Administration

Establishing a functioning consortium requires buy-in and a high level of cooperation from all partners. Prior to initiating programming, all potential institutional representatives (Graduate, Research, Postdoc, Faculty Affairs and/or Diversity Deans and senior administrators (2 to 3 per institution)) from the Big Ten Academic Alliance Consortium set initial goals to: i.) address the campus needs for mentor-up skill development for postdocs and mentor facilitator training for staff and faculty; ii.) establish sustainable communities of practice for mentor training; and iii.) develop mechanisms for central coordination, outreach to campus constituents, templates for recruitment of participants, and strategies to sustain collaboration. Recruitment of participants was also accomplished via these campus representatives. Continuity in communication was maintained by establishment of an Executive Committee, monthly conference calls, team meetings, and most importantly, attendance of the campus representatives as observers or facilitators at all mentor training venues, with immediate group feedback. Although not uniform across all institutions, active participation during the entire 4 year period was strong (average 35 faculty/administrators and 25 postdocs/year) at each session (capped by Master Facilitators), which was essential for establishment of the infrastructure needed for implementation back on the home campuses, and continuity in reporting outcomes. Big Ten Academic Alliance/NRMN-CAN members included: Michigan State University, Pennsylvania State University, Indiana University, Northwestern University, Ohio State University, Purdue University, University of Minnesota, University of Chicago (affiliate member), University of Michigan, University of Illinois at Chicago (affiliate member), University of Illinois at Urbana-Champaign, University of Wisconsin-Madison, University of Iowa, University of Nebraska-Lincoln, Rutgers University-New Brunswick, and University of Maryland.

### Curricula

In order to help postdocs get the most out of their mentee relationships as well as gain mentoring skills for the future, the “Mentoring-Up” curriculum [14], which focuses on defining and articulating the factors that build a sense of self-efficacy, i.e., past accomplishments, vicarious modeling, social persuasion, and positive-affected states as related to postdocs’ dual roles as mentees and mentors was implemented for postdocs from the Big Ten Academic Alliance by NRMN Master Facilitators (https://nrmnet.net/research-mentor-training/) at the Big Ten Conference Center. “Mentoring-Up” is adapted from the concept of “managing up” [35] and incorporates and adapts training curriculum from *Entering Mentoring* [28] and *Entering Research* [36] curricula. The seven Core Principles are: 1. Two-way communication, 2. Aligning expectations, 3. Assessing understanding, 4. Fostering independence, 5. Ethics, 6. Addressing equity and inclusion, 7. Promoting professional development.

This curriculum provided postdocs opportunities for: i.) self-evaluation and reflection to become aware of their personal biases, attitudes, and behaviors; ii.) exploring strengths, weaknesses, and challenges in their interpersonal and professional relationships; iii.) understanding and learning how to use the mentor principles; and iv.) focusing on cognitive processes that may lead to behavioral changes and strategies to facilitate those changes in a process-based approach over 1.5–2 day workshops (typical schedules are provided in Supplement 1). The learning objectives were addressed with activities in small-group discussions to: share experiences; read and discuss research studies on strategies for communicating across disciplines, generations, ethnicities, positions, etc.; these activities were interspersed with relevant case studies to stimulate thinking about the elements of good mentoring relationships and developing strategies for overcoming challenges and creating opportunities. Each module of the curriculum was considered from the dual perspective of the postdoc as current mentee and as a future mentor. This was a powerful approach to help postdocs internalize the training concepts, translate them into increased awareness, and hopefully improve their relationships as they transition along their professional career paths.

Faculty and senior administrators from across the Big Ten Academic Alliance were also trained by NRMN Master Facilitators (https://nrmnet.net/master-facilitator-bios/) at the Big Ten Conference Center to subsequently run mentor-training workshops on their own campuses. The process-based curriculum was adapted from *Entering Mentoring* [33], which focuses on six key competencies: 1. maintaining effective communication; 2. establishing and aligning expectations; 3. assessing understanding of scientific research; 4. addressing diversity; 5. fostering independence; and 6. promoting professional development. The 1.5–2 day workshops (schedule provided in Supplement 2) included case studies and activities that: i.) engage mentors in peer discussion of a mentor framework; ii.) explore strategies to improve mentoring relationships; iii.) address mentoring problems; iv.) reflect on mentoring philosophies; v.) and create mentoring action plans to model the interactive, collaborative, and problem-solving ways to develop and implement this set of trainings in the future. The training goals provided tools and mechanisms to implement mentor training venues at the participating institutions, thereby establishing sustainable Mentor-training programs for undergrads, graduate students, postdocs and faculty. Note: The postdoc and facilitator training sessions were held in the same facility (Big Ten Conference Center) and occurred separately but concurrently. Postdocs, faculty and administrator participants networked during breaks, etc.

### Data collection and evaluation

Over the course of this project, 102 postdocs, and 160 faculty and senior administrator mentor-facilitators participated in NRMN-CAN Mentor training. Postdocs and trainers recruited by campus representatives created a personal profile on the NRMN website (NRMNet.net) and completed an online registration form specific to the NRMN-CAN workshop. For Postdoc “Mentoring-Up” and faculty/administrator Facilitator training, evaluation surveys (Satisfaction and Competency) were administered to participants pre-and post-training via Qualtrics Survey Software and analyzed by the NRMN Mentor Training Core. A specific NRMN-CAN survey was developed and implemented in Spring 2019 for all four postdoc cohorts to ascertain whether mentor training: i.) influenced career progression; ii.) impacted the postdocs’ relationship with their PIs; and iii.) components of the mentor training that were implemented by the postdoc mentees. For responses other than demographic data, postdocs: i.) selected options on a 7-point Likert-type scale (extremely positive to extremely negative), and ii.) followed by ‘why’ or ‘how’ questions. A dedicated NRMN-CAN survey for faculty and senior administrators was also developed to ascertain whether participation in Mentor Facilitator training led to: i.) implementation of training workshops on their campuses; ii.) the level and number of participants; iii.) and whether facilitated sessions were carried out in partnership with others. In order to increase response rates, the NRMN-CAN Administrative Core reached out personally via email or phone contact to every participant and campus representatives for periodic updates. Data were collected up to May 2019 and individual responses for both surveys were collected into a secure REDCap database (IRB Protocol Number 15–1724 at the University of Chicago).

### Campus awards

In order to catalyze Mentor training programs at member institutions, a NRMN-CAN Campus Awards Program was initiated in the last two years, once a pool of trained facilitators was established. Twelve awards were competitively distributed and ranged from $1500 to $2500 per award.

## Results

Data collected and reported below describe the demographics and workshop evaluation surveys from the 2016–2019 postdoc workshop participants/cohorts as well as the faculty mentor training groups.

### Demographics of the postdoc participants

Between Spring 2016 and Spring 2019, 102 postdocs (average 25 per year) from 14 of the 16 Big Ten Academic Alliance institutions participated in “Mentoring-Up” training. The NRMN-CAN collaborative approach was successful in bringing together an ethnically and racially diverse population of postdocs (63% UR); all were U.S. citizens or permanent residents; women comprised 68% of participants; 97% had a Ph.D. as the terminal degree; the rest had professional degrees (M.D. or D.V.M) or dual degrees (Ph.D. and M.D. or D.V.M); research foci included laboratory-based (51%), clinical (7%), social/behavioral (24%), and public health/community (19%) (Table 1).

**Table 1:** Postdoc mentor training demographics, 2015–2019.

Postdoc Characteristics	Postdoc Participants		Survey Respondents	
	**#**	**%**	**#**	**%**
**Total Participants**	102	100%	69	68%
**Gender**				
Male	33	32%	20	29%
Female	69	68%	49	71%
**Race/Ethnicity**				
White	28	27%	21	30%
Asian	10	10%	6	9%
African American+	35	34%	27	39%
AI, AN, PI, NH*	4	4%	1	1%
Hispanic or Latino	23	23%	16	23%
Other (multi-racial)	2	2%	1	1%
**Total UR**	**64**	**63%**	**45**	**61%**
**Degree**				
PhD	99	97%	67	97%
MD or DVM	1	1%	1	1%
PhD and MD or DVM	2	2%	1	1%
**Research Focus**				
Laboratory	52	51%	35	51%
Clinical	7	7%	8	12%
Behavioral	24	24%	19	28%
Community	19	19%	7	10%

Table 1 summarizes the self-reported demographics and professional characteristics of the 102 postdocs who participated in the four NRMN-CAN “Mentoring-Up” workshops held between 2016 and 2019, along with the profiles of the 69 postdocs who completed the NRMN-CAN follow-up survey (68% response rate). The UR designation includes postdocs from racial or ethnic backgrounds defined by NIH to be underrepresented in the biomedical science workforce, including Hispanic or Latino, Black or African American, American Indian, Alaskan Native, Pacific Islander or Native Hawaiian, or multi-racial that includes at least one of these backgrounds. These data show that the characteristics of the survey respondents were representative of those of the postdoc participants.

*American Indian, Alaskan Native, Pacific Islander or Native Hawaiian

+ One postdoc with a physical disability is included in this participant group

Postdocs were then surveyed 1–3 years after the NRMN-CAN “Mentoring-Up” workshops, in order to assess whether the training helped them to more effectively guide the mentoring they received as current mentees and to better prepare them to be future mentors. Of the 102 postdocs who received Mentor training between 2016 and 2019, 69 (68%) responded, including: 71% female, consistent with the gender distribution (68%) of all participants; and the majority of participants and survey respondents were predominantly underrepresented in STEM, 63% and 61%, respectively. The gender of their primary mentor was nearly equally distributed between male and female (48.5% and 51.5%, respectively), in contrast to a previous study [16], which skewed toward male mentors (70%). Career sectors tracked between participants and respondents with about 50% in lab-based research, 12% in clinical, 28% in behavioral and 10% in public health/community-based research (Table 1). By these demographic metrics, the survey respondents largely reflected the workshop participant characteristics.

### Evaluation of Postdoc’s relationship with mentor

**Fig 1:**
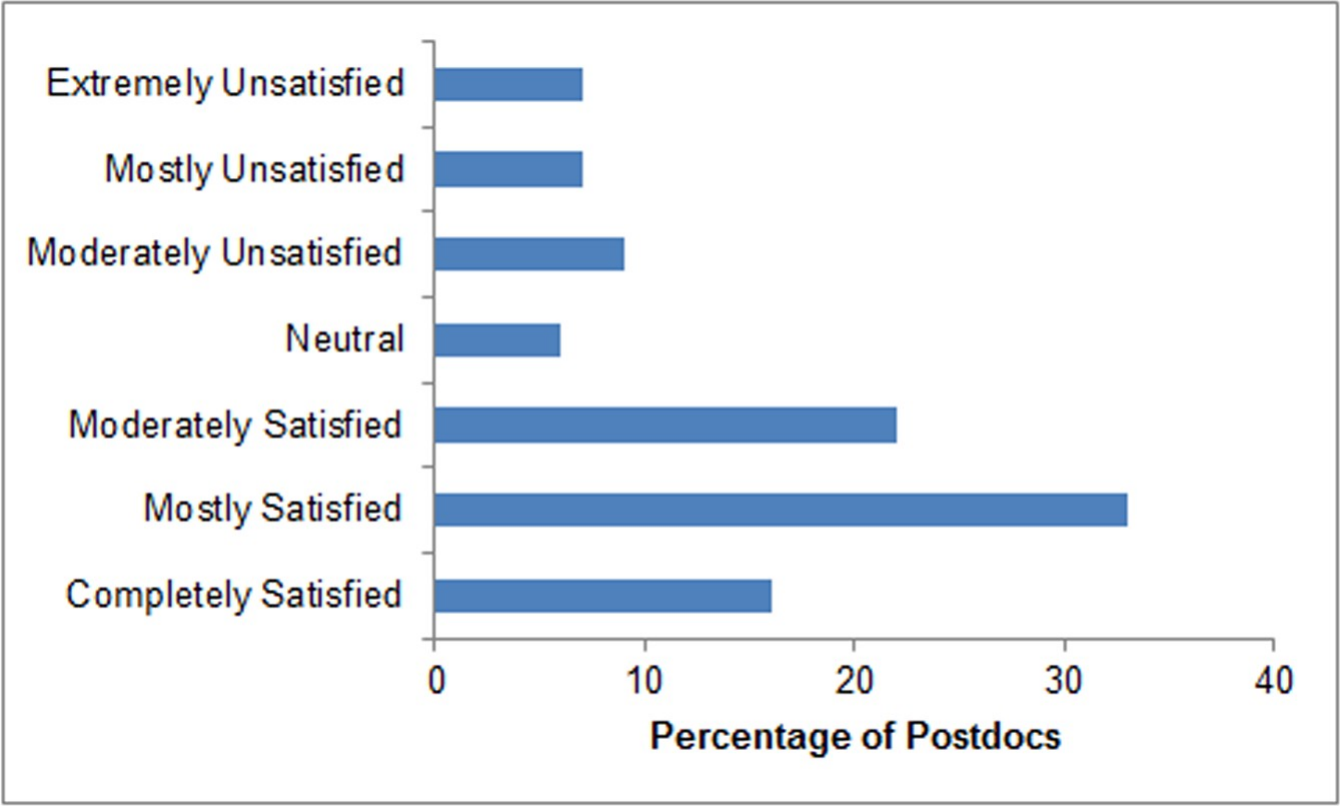
Overall postdoc mentor satisfaction. Surveyed NRMN-CAN postdocs were asked to rate their level of overall satisfaction with their postdoctoral mentor on a 7-point Likert scale. Results are shown as percentages of postdoc responses.

**Fig 2:**
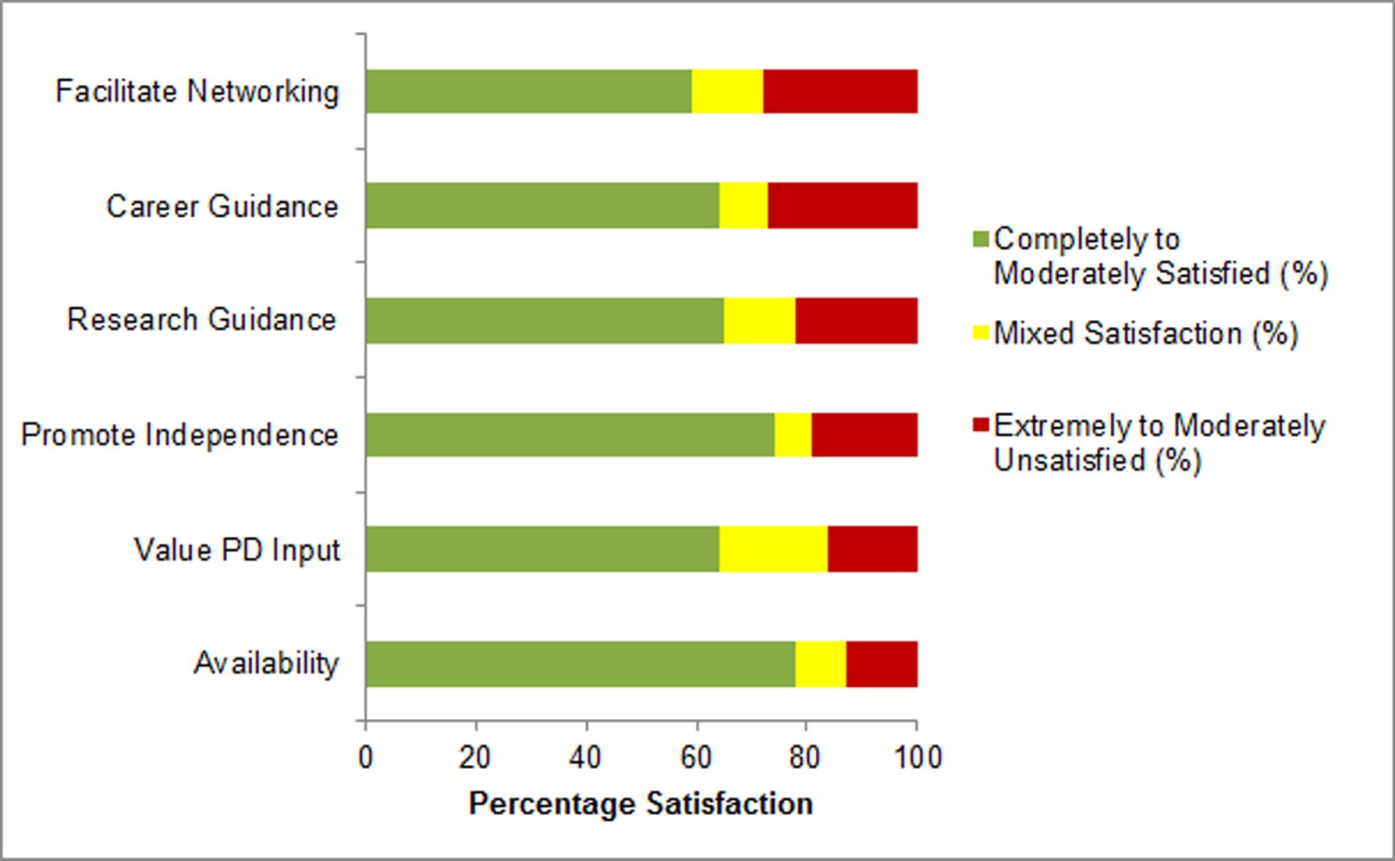
Postdoc satisfaction with specific attributes of their mentor. NRMN-CAN postdocs were asked to rate satisfaction levels for several attributes of their postdoc mentor on a 7-point Likert scale (completely satisfied, mostly satisfied, moderately satisfied, mixed satisfaction, moderately unsatisfied, mostly unsatisfied, or extremely unsatisfied). For ease of presentation, *Completely to Moderately Satisfied* represents the three “satisfied” categories, *Mixed Satisfaction* represented the middle rating, and *Extremely to Moderately Unsatisfied* represents the three “unsatisfied” categories.

One of the goals of the “Mentoring-Up” training is to improve the postdocs’ relationship as mentees with their PIs/mentors. A combination of quantitative and qualitative data were collected from the surveys to provide a broader view of the participants’ experiences. When queried with respect to outcomes of mentor training, 71% of respondents were either completely, mostly or moderately satisfied with their postdoc mentor (Fig 1). High overall satisfaction (59–78%) was also mirrored in most desired mentor attributes (i.e., availability, research and career guidance, and promote independence). However, importantly, 28% of the postdocs rated their postdoc mentor’s provision of career guidance and facilitating networking as moderately to extremely unsatisfactory (Fig 2).

**Fig 3:**
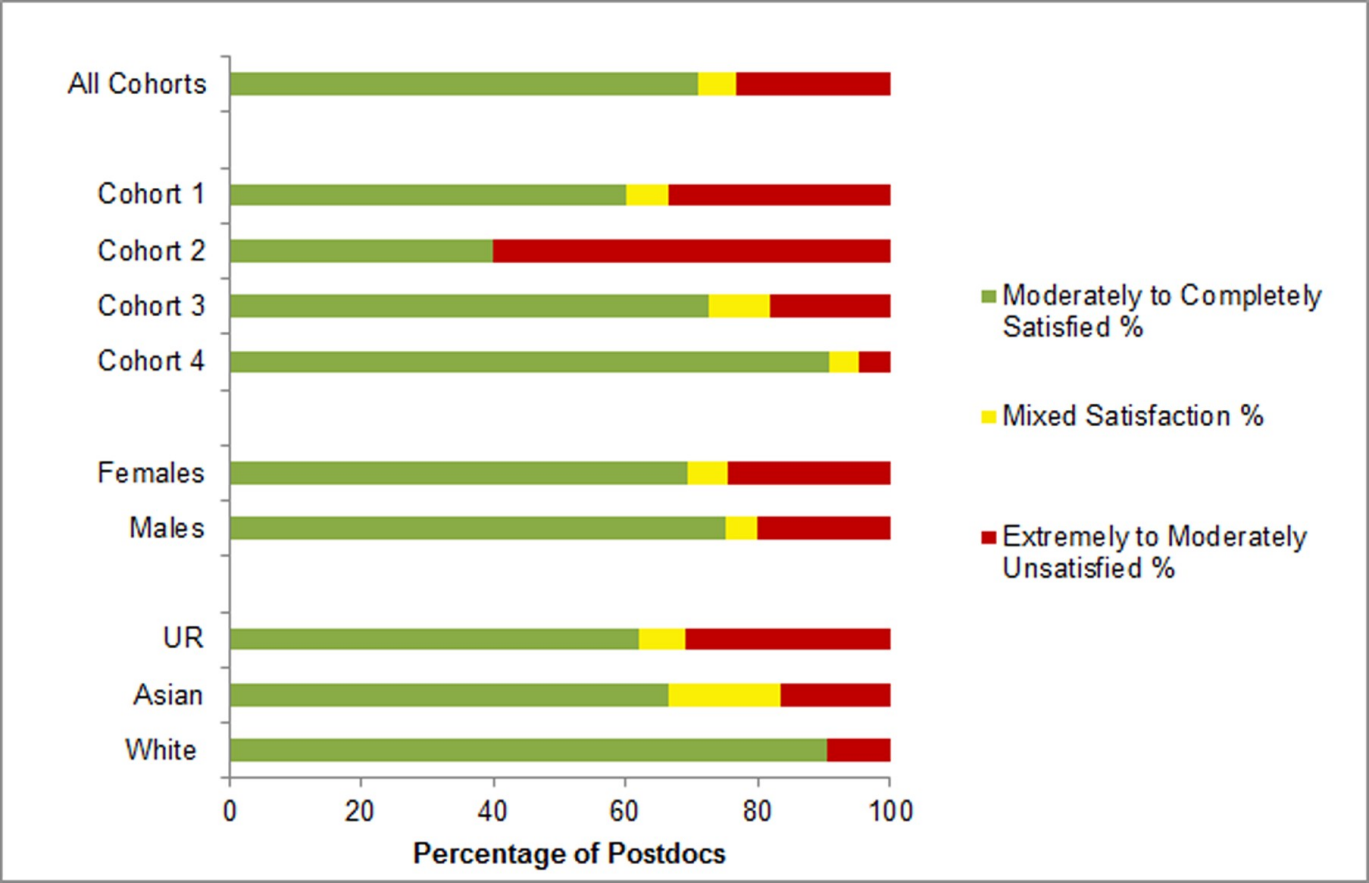
Postdoc characteristics and overall satisfaction with their mentor. Surveyed NRMN-CAN postdocs were asked to rate their level of overall satisfaction with their postdoctoral Mentor on a 7-point Likert scale, and results are shown as the average rating for all postdoc respondents, then separated as averages for each of the four cohorts and for different demographic categories of postdocs. S*atisfied* and *Unsatisfied* responses were combined as described in the legend for Fig 2. The UR designation includes postdocs from racial or ethnic backgrounds defined by NIH to be underrepresented in the biomedical science workforce. As presented in Table 1, for this program, these categories include Hispanic or Latino, Black or African American, American Indian, Alaskan Native, Pacific Islander or Native Hawaiian, or multi-racial that includes at least one of these backgrounds.

**Fig 4:**
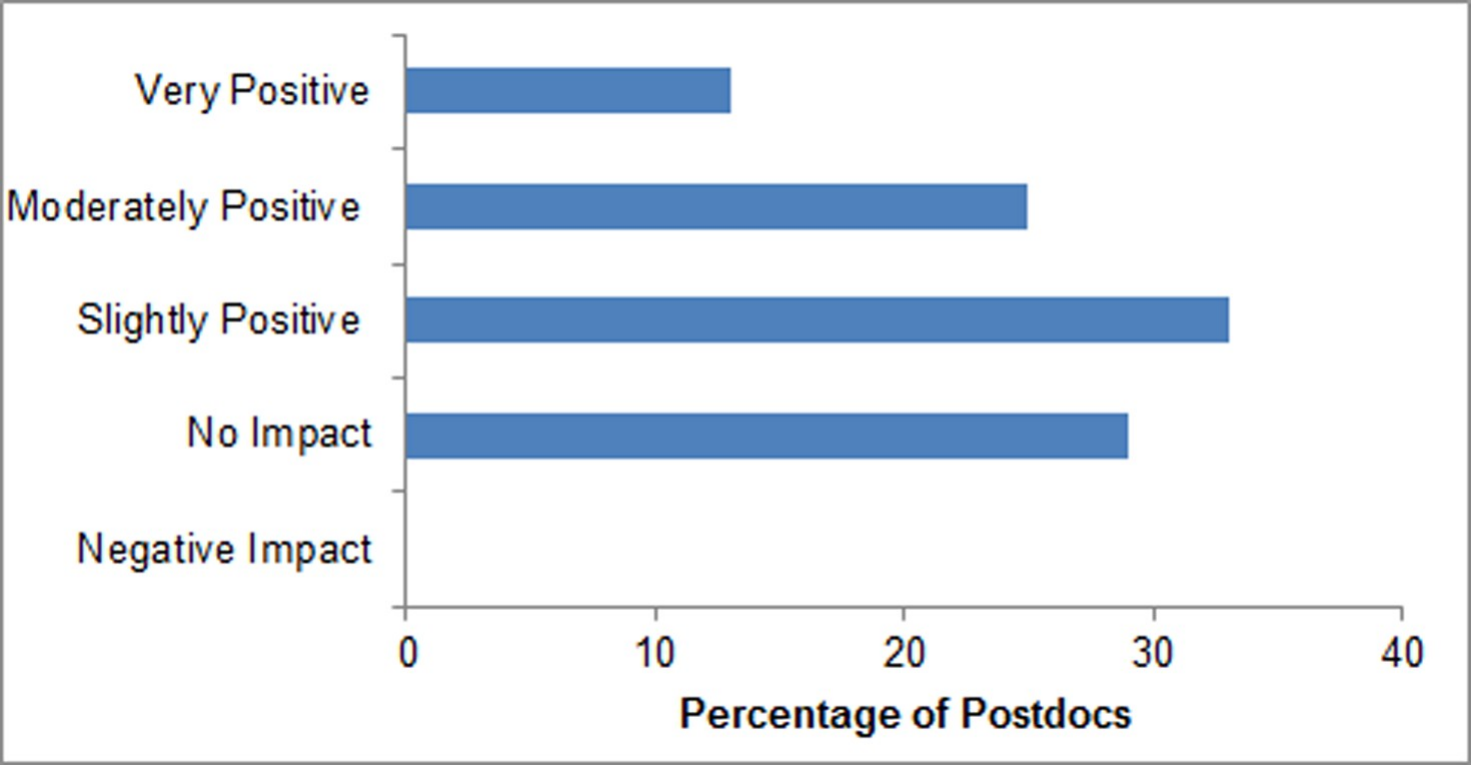
Mentor training impact on relationship with mentor. Postdoc participants were asked to report on their satisfaction after the NRMN-CAN “Mentoring-Up” workshop on their relationship with their postdoc mentor, based on a 7-point Likert scale: very negative impact, moderate negative impact, slight negative impact, no impact at all, slight positive impact, moderate positive impact, or very positive impact. No postdoc reported slight, moderate or very negative impacts, which were combined in the *Negative Impact* column in the Figure.

Interestingly, satisfaction/dissatisfaction with PI/Mentor trended toward increasingly positive responses as postdocs participated more recently in mentor training, with the latest cohort from 2019 reporting the highest satisfaction levels with the mentoring they received from their PIs (Fig 3). A large proportion of both males (75%) and females (69%) were moderately- to completely- satisfied with mentoring from their PIs (Fig 3). UR (62%) and Asian (67%) postdocs were somewhat less satisfied with their mentoring compared to white postdocs (90%). Underrepresented (31%) postdocs compared to white (10%) or Asian (17%) postdocs, were more dissatisfied with their mentors (Fig 3). In all cohorts, over 70% of the postdocs felt that mentor training moderately- to- very-positively impacted their relationship with their current mentors, while 29% indicated mentor training had no impact, but importantly **no** postdoc indicated that the training had a negative impact (Fig 4).

In order to determine whether postdocs gain knowledge and skills needed to improve their mentoring practices as a mentee, postdocs were asked to expound on the effects this training and the related discussions had on their relationships with their mentors. Their written responses (summarized in Table 2) fell into five main categories: i.) improved postdocs’ confidence/courage/self-efficacy/validation to speak up, take control, be more pro-active; ii.) developed new communication strategies by the postdocs to manage the relationship, align expectations, have difficult conversations and execute more effective negotiations; iii.) enhanced the postdoc’s perception of their role as a mentee overall by seeing the relationship from the other side; iv.) improved the relationship with the PI (mentor) as well as the research environment for others indirectly by sharing highlights, expectations, compacts, and other resources from the workshops with receptive mentors; (as an example, one mentor asked the postdoc to present lessons learned in the “Mentoring-Up” workshop to the rest of their lab group!); and v) empowered the postdoc to critically re-evaluate the relationship with their PI and align expectations for independence (Table 2).

**Table 2:** Informative survey responses for how mentor training helped postdocs to be better mentees.

Category	Survey Quotes
**1. Improved postdocs’ confidence/courage/self-efficacy/ validation to speak up, take control, be more pro-active.**	“It is much easier to discuss long-term research and career goals with my mentor”
	“The workshops taught me ways to communicate with my PI and that it is ok to make my views of how I wanted to be mentored by my PI. Initially, I thought I should just take what I got from them and didn’t have a voice in the matter.”
	“The session has increased my self-efficacy and gave me more confidence to guide my own research and career development.”
	“As a female in a male-dominated field, I am more confident and authentic to do things ‘my way’.”
	“I am much more confident in communicating my needs as a mentee.”
	“The workshops gave me confidence and empowered me to take more control of my career trajectory.”
	“I have more courage to speak up and tell him how he can help me.”
	“It affirmed I could be a successful future mentor and academic PI.”
	“Daily communication has improved.”
	“I am now more pro-active about what I want.”
**2. Provided strategies for the postdocs to manage the relationship, have difficult conversations and execute more effective negotiations.**	“The workshop helped in aligning expectations and promoting more honest communication.”
	“I am using the strategies discussed in the workshops to evoke connections with my PI on career development.”
	“It helped put things in perspective and gave me skills to facilitate discussion and negotiations using more powerful language and wording instead of vague ‘wants and needs’.”
	“It demonstrated how to have difficult conversations with my mentor.”
	“It changed how I communicate my professional goals and needs.”
	“It provided strategies to make the time with my mentor more efficient and effective.”
	“I was able to open up to my mentor and make him aware of methods that work for me.”
	“I have a good relationship with my mentor as she treats me as a colleague, but still some of the take-aways were valuable.”
	“I left the workshop with a mindset to be more intentional, maximizing my experience as a mentee.”
**3. Enhanced the postdoc’s role as a mentee overall by seeing the relationship from the other side.**	“I was able to be a better mentee by seeing the relationship from the other side.”
	“We are working on aligning expectations and having more honest communications.”
**4. Improved the relationship with the PI and the research environment for others indirectly by sharing highlights, expectations, compacts, and other resources from the workshops with receptive mentors.**	“I shared my experience and highlights of the workshops with my mentor–it provided an opportunity to talk about areas to improve that we had not previously considered.”
	“I showed my mentor the expectations/contracts/compacts discussed in the workshops, which are fostering better communication and research productivity.”
	“My relationship with my mentor improved significantly because I implemented the strategies I learned to manage the aspects of our relationship I was not happy with.”
	“My mentor asked me to present the lessons learned to our lab group.”
	“Our relationship has remained largely unchanged although he did voice interest in discussing career paths recently.”
**5. Empowered the postdoc to re-evaluate the relationship with the PI.**	“I decided to switch postdoc mentors in order to aid in the career guidance I wanted.”
	“I tried to implement some of the skills learned from the workshop, but it didn’t end up changing how my postdoc mentor treated me.”
	“The workshops made me aware of what I was lacking in my relationship with my mentor, and made me realize I can only compromise so much to gain value from the relationship”
	“After the workshop, I left the postdoc because it validated my dissatisfaction.”

The qualitative data indicate significant improvements in postdoc mentee-mentor relationships as a result of attending a NRMN-CAN “Mentoring-Up” workshop. One postdoc reported “My relationship with my mentor improved significantly because I implemented the strategies I learned to manage the aspects of our relationship I was not happy with.” Another postdoc who already had a good mentoring relationship reported “I have a good relationship with my mentor as she treats me as a colleague, but still some of the take-aways were valuable.” Perhaps one of the best take-aways for postdocs was their improved communication skills as captured in this postdoc’s comments “It helped put things in perspective and gave me skills to facilitate discussion and negotiations with more powerful language and wording instead of vague “wants” and “needs”.” Some comments were less clear to interpret but had some encouraging words “Our relationship has remained largely unchanged although he did voice interest in discussing career paths recently.”

### Evaluation of mentoring-up training on postdocs’ perception of their mentoring skills

We also inquired whether postdocs implemented components of the training with their mentees and how “Mentoring-Up” training impacted their relationship with their mentees (undergraduate or graduate students, etc.). An overwhelming proportion (87%) of postdocs indicated that the training was helpful in improving their mentoring proficiency and they had already used the skills learned in the workshops (Table 3). It should be noted that for most of these postdocs (69%), this was the only mentor training venue in which they participated, with both UR (74%) and female (71%) postdocs most likely to have had no additional mentor training. For the approximately 30% of postdocs who had additional mentor training, it was predominantly (76%) at their home institution. Interestingly, female participants were more likely (92%) than males (75%) to report that they implemented mentoring skills they had learned (Table 3).

**Table 3:** Postdoc mentor training characteristics and outcomes.

Postdoc Characteristics	Implemented Mentor Training Skills for Mentees (%)			Had Additional Mentor Training (%)		
**Gender**	**Total Responses**	**Yes**	**No**	**Total Responses**	**Yes**	**No**
**Female**	71	92	8	71	29	71
**Male**	29	75	25	29	35	65
**Demographics**	** **			** **		
**UR**	61	88	12	62	26	74
**White**	30	90	10	31	38	62
**Asian**	9	67	33	7	40	60
**Total**	**100**	**87**	**13**	**100**	**31**	**69**

In Table 3, postdoc survey responses, by demographic and gender groups and in total, are shown as percentages of respondents. These data are in response to survey questions regarding whether the participants implemented what they had learned at the “Mentoring-Up” workshop in their own mentoring relationships with their mentees, and whether they attended any additional mentor training workshops after the NRMN-CAN sessions.

When postdocs were asked to comment on the effects “Mentoring-Up” training had on their mentoring skills and how it helped to improve their mentoring practices with their mentees, their responses fell into five domains: i.) recognizing the joy and satisfaction that meaningful mentoring can have as a Mentor; ii.) reflecting more intentionally on mentees’ strengths, needs and goals (assessing understanding), and how to instill self-efficacy and confidence in their mentee’s abilities (promoting independence); iii.) providing invaluable tools and resources that improved mentoring skills; iv.) sharing knowledge and tools with others and using the skills learned as the postdocs’ careers progress; v.) impacting future planning, goal setting and community building (Table 4). For example, one postdoc reported “The mentor training helped me tremendously and encouraged me to take on an undergrad, which I had no confidence to do before.” By taking on new responsibilities, this postdoc’s career progressed as more independence and confidence was the result of training. Another post-doc reported “The mentoring workshop helped me to take a step back and evaluate my mentoring style to identify strengths and areas of potential growth. I feel more confident in my ability to be an impactful mentor and cultivate self-efficacy within my mentees.” One of the best take-aways for postdocs was the joy garnered from mentoring others: “Making an impact on trainees’ lives is rewarding and NRMN helped me become more aware and savor the experience.”

**Table 4:** Informative responses for how the mentor training has helped postdocs be better mentors.

Category	Survey Quotes
**1. Recognizing the joy and satisfaction that meaningful mentoring can have as a Mentor.**	“I have had outstanding mentees who were a joy to work with, several of whom have moved onto med/grad school, sending back beautiful thank you notes.”
	“Making an impact on trainees’ lives is rewarding and NRMN helped me become more aware and savor the experience.”
	“The workshops taught me skills on how to maximize the benefits of the mentee relationship for both myself and my mentees.”
	“I am much more involved with my mentees and enjoyed it more!”
	“I am listening more to my mentees and try to suggest strategies that worked for me.”
	“Being intentional with my mentees about their goals and creating space for them to articulate what that means to them.”
	“The most important take away for me was to create an environment so that your mentee feels free to communicate fears, confusion, and joys to me.”
**2. Reflecting more intentionally on mentees’ strengths, needs and goals, and how to instill self-efficacy and confidence in their mentee’s abilities.**	“I now more readily recognize the strengths of my mentees, rather than only the deficiencies.”
	“I learned to approach mentoring more strategically and develop mentee-specific goals and metrics.”
	“I have changed the methods I used previously to provide feedback to mentees as I now recognize the importance of using more positive approaches/language to be more constructive with feedback.”
	“I spend more time contemplating how to effectively build up mentees’ self-efficacy and confidence to succeed in their academic undertakings.”
	“I am more concerned with how I am promoting my mentees development.”
	“I try to motivate the mentees based on their interests and desire to be mentored.”
	“I feel I can instill confidence and self-efficacy in my mentees now.”
	“It made me more aware of my mentees’ communication styles and needs.”
	“As a result of the case study discussions I am more cognizant of mentees different cultural backgrounds.”
**3. Providing invaluable tools and resources that improved mentoring skills to set expectations and improve communication.**	“The mentor training helped me tremendously and encouraged me to take on an undergrad, which I had no confidence to do before.”
	“I now know how important it is to set clear expectations upfront, to be flexible and maintain open communication.”
	“I am using the mentoring contracts and other resources as tools in my mentee relationships.”
	“I am clearer in articulating expectations and goals.”
	“It gave me valuable tools for providing positive feedback.”
	“I learned skills to facilitate productive relationships with mentees from the beginning.”
	“At the beginning of working with a new mentee, we align expectations and use a ‘compact’ for the training period.”
	“I use the guidelines to support students’ goals and expectations.”
	“Definitely so! I gained insights to set goals and expectations in guiding my mentees.”
	“I gained clarity about strategies, and never thought about these issues and how important it is to build the relationship in a mutually beneficial, productive way.”
	“Immediately after the workshop, I changed my work interactions with my undergrad.”
	“It helped me to identify areas where I need to improve, but also helped me recognize the mentoring aspects I have been doing well.”
	“It gave me the tools to be a more impactful mentor.”
	“I feel more equipped to ‘mentor down’.”
	“Setting tone for honest/trusting relationship.”
	“I am more mindful and systematic in my behavior and expectation with my mentees.”
	“I think deeper about the importance of communicating throughout the mentoring process.”
**4. Sharing both knowledge and tools with others and using skills learned as postdocs’ careers progress.**	“I shared what I learned with my lab and we will change our approach with summer students.”
	“I have shared the curriculum and resources with other postdocs as they work with undergrads.”
	“I have integrated many of the components of the training and we are planning to organize workshops to disseminate what we learned to others on campus.”
	“I have shared the resources with mentees and mentors at other institutions so they can benefit as I have.”
	“I won ‘Mentor of the Year’.”
	“Although I have left academia, I am using the lessons I learned as both a mentor and mentee in industry.”
	“The training was quite helpful and my interactions with students improved a lot from the training. As a professor now, I used the tools every day.”
**5. Impacting future planning, goal setting and community building.**	“It opened new ways of approaching my future role as a mentor when I transition to an Assistant Professor position this fall.”
	“I was on the job market during the workshop and it helped me identify and shape the type of mentor/assistant professor I wanted to be with my students.”
	“It provided space to think critically about mentoring and how best to use this postdoc step.”
	“The training provided an avenue to reflect on my current mentor-mentee relationship with my students and principal investigator.”
	“It afforded me the opportunity to look forward to my future role mentoring all tiers of students.”
	“I am more confident by having help and understanding that others have gone through similar experiences and can guide me with examples of how they overcame challenges.”
	“The NRMN-CAN workshops provided a new and more inclusive environment in which to explore these mentoring skills.”
	“It provided space to meet postdocs from different backgrounds and fields–an opportunity I rarely had as a postdoc.”
	“Helped me realize that concerns and challenges are common at this stage and, importantly, not unique to me.”
	“Overall I feel more confident and that I belong. I still communicate with the mentors I met through NRMN-CAN.”

### Influence of mentor training on developing independence and moving forward on career path

The majority of the respondents (80%) across all demographics (Table 5) indicated their long-term career goals were as academic faculty-primarily research/teaching. Most (59%) felt that the mentor training influenced their career decisions and next steps, especially with respect to pursuit of an academic career; females (59%), UR (62%) and Asian (67%) indicated a strong positive influence. Thus far, 29% of the respondents who participated in mentor training as postdocs have transitioned to faculty positions (Table 5). These include 85% UR, 75% females, and 65% at Big Ten Academic Alliance institutions.

**Table 5:** Impact of mentor training on postdocs career goals and outcomes.

Postdoc Characteristics	Total Responses (%)	Career Goals (%)		Influence of Mentor Training on Career Plans (%)		Transition to Faculty (%)	
Gender		Academic	Other	Yes	No	Yes	No
Female	71	78	22	59	41	31	69
Male	29	85	15	60	40	25	75
**Demographics**							
UR	61	79	21	62	38	40	60
White	30	81	19	52	48	10	90
Asian	9	83	17	67	33	17	83
**Total**	**100**	**80**	**20**	**59**	**41**	29*	**71**

In Table 5, total survey results by demographic and gender category are shown as percentages of respondents. Postdocs were asked to indicate their primary career goals, and whether attending “Mentoring-Up” had any influence on their career plans. In the final column, the percentage of postdoc participants from the “Mentoring-Up” program who have successfully been appointed to faculty positions is shown.

*Of those individuals who transitioned to faculty positions, 85% were UR and 75% female.

Many postdocs also indicated that mentor training has made it easier to adapt to and assume the responsibilities of being a faculty mentor, and several indicated that their mentor training and experiences were valuable criteria when applying for faculty positions (Table 4): “Mentor training was definitely a plus when applying for faculty positions”.

One postdoc explained how the workshop had impacted their job search: “I was on the job market during the workshop and it helped me identify and shape the type of mentor/assistant professor I want to be with my students.” Reflection was important to some postdocs: “It provided the space to think critically about mentoring and how to best use this postdoc step.” Another stated: “The training provided an avenue to reflect on my current mentor-mentee relationship with my students and principal investigator.” “It afforded me the opportunity to look forward to my future role mentoring all tiers of students.” In addition, the discussions and networking opportunities with other postdocs with similar interests was important; especially for some postdocs of color, who are often isolated at their home institution: “The NRMN-CAN workshops provided a new and more inclusive environment in which to explore these mentoring skills.”

### Institutional capacity-building and sustainability

In addition to aiding predominantly UR postdocs to gain mentoring skills necessary to enter and succeed in an academic career, a second major goal of this initiative was to increase the critical mass of mentor facilitators and build institutional capacity for mentor training. This goal was accomplished by creating cadres of faculty and institutional leaders who developed core competencies in mentoring, so that trainee- and faculty- mentor training venues could be implemented and sustained on partner campuses.

Mentor Facilitator training was completed between 2016–2019 with 160 total participants; 115 (72%) faculty and 45 (28%) senior administrators, which included 151 members from the NRMN-CAN/Big Ten Academic Alliance institutions, and 9 guests from Predominantly Undergraduate Institutions (PUI) or UR-serving institutions that have strong relationships with Big Ten Academic Alliance schools around Chicago or the East Coast. As well, 20 new or returning participants have undertaken the Culturally Aware Mentor (CAM) training, a recently developed second-level workshop focused on diversity [34]. The 160 participants represented a broad spectrum of backgrounds: 66% female; 28% UR; 81% Ph.D.s; 9% M.D.s or M.D./Ph.D.s; 7% M.S. or M.P.H.; 3% DPharm. (Table 6); 15 (10%) were Deans or Associate Deans of Diversity, Graduate Education, Postdoc -, Research- or Faculty- Affairs; and 6 (4%) were training grant directors. The inclusion of senior administrators at the NRMN-CAN trainings proved to be essential for implementing subsequent mentor training workshops on their home campuses especially with respect to many of the previously identified tangible barriers [29], based on follow up conversations with these participants.

**Table 6:** NRMN-CAN faculty/staff facilitator mentor training participant demographics, 2016–2019.

Total Faculty & Staff Participants	#	%
	160	100%
**Gender**		
Male	55	34%
Female	105	66%
**Race/Ethnicity**		
White	87	54%
Asian	18	11%
African American	23	14%
AI, AN, NH, PI*	4	3%
Hispanic	14	9%
Other or multi-racial	4	3%
Not reported	10	6%
**Position**		
Faculty	115	72%
Senior Staff	45	28%
**Degree**		
PhD	130	81%
MD or MD/PhD	14	9%
MS or MPH	11	7%
PharmD	5	3%

Table 6 summarizes the self-reported demographics and professional characteristics of the 160 faculty and staff who participated in the four NRMN-CAN Facilitating Mentor Training workshops held between 2016 and 2019.

*American Indian, Alaskan Native, Pacific Islander or Native Hawaiian

In order to determine the impact and potential sustainability of mentor-facilitator training at member campuses, participants in NRMN training between 2016 and 2019 were surveyed. As shown in Table 7, progress toward this goal has been substantial. Thus far, of the 151 Big Ten facilitators who have been trained (range 2 to 26 per campus), 49% (59 out of 120 from the first three years) have implemented mentor training opportunities on their campuses that reached 2,560 graduate students, postdocs, and faculty (Table 7). An additional 40% (16 out of 40 from the Spring 2019 workshop) indicated that they would be implementing mentor training in the upcoming months (data collection was terminated May 2019 and the last training session was held in March 2019). Interestingly, of those that have implemented mentor training, 90% responded that they facilitated with others; 20% with faculty colleagues, 21% with administrators, and 50% with both faculty and administrators in teams.

**Table 7:** Mentor training on NRMN-CAN consortium campuses.

Institution	# NRMN-CAN Mentor Facilitators	# Campus Participants
1*	27	680
2*	18	85
3*	16	463
4	14	6
5*	12	410
6*	11	230
7*	11	54
8	9	83
9*	9	77
10	8	26
11	5	180
12*	4	66
13	3	0
14	2	200
15	2	0
16	0	0
**Total #**	**151**	**2560**

Table 7 indicates the numbers of faculty and staff participants who were trained to facilitate mentor training workshops per Big Ten Academic Alliance institution, along with the reported number of campus participants who attended subsequent mentor training workshops led by these facilitators. Excluded were the 9 trained participants from guest institutions outside of the Big Ten Academic Alliance.

*Received an NRMN-CAN Campus Award to fund trainings on campus.

### Campus awards

Unlike the Mentor Facilitator training which brought people from all campuses to the Big Ten Conference Center for training, institutional professional development and mentor training on individual campuses was further promoted via an innovative NRMN-CAN Campus Awards Program that provided twelve competitive awards (6 in 2018, and 6 in 2019). As documented in the application for the Campus Award, there was no other source of funding on their home campus, and therefore the training opportunity would not have proceeded without the Award. Sessions were organized by institutional representatives and programming was executed by faculty mentor-facilitator alumni of the NRMN-CAN consortium workshops or outside experts. Content topics included: “Optimizing Mentoring Relations,” “Developing Dual Mentor/Mentee Skills,” “Mentoring-Up,” and “Putting Mentoring at the Heart of Academia,” culminating in June 2019 with the Future of Research Symposium on “Mentoring Future Scientists” co-sponsored by NRMN-CAN. Several of these offerings included professional development topics to help mitigate the lack of career guidance by PIs. In addition, one institution provided mentor training for 130 faculty, postdocs and graduate students prior to their 2019 summer research program and also produced videos for follow-up training activities: https://vimeo.com/338750055 and https://vimeo.com/274766897. Collectively, an additional 401 graduate students, postdocs, and junior faculty have benefitted from this novel and cost-effective approach. As well, these workshops fostered opportunities for local networking across disciplines, exposure to institutional and external experts and resources, and inclusive community-building.

## Discussion

**Fig 5:**
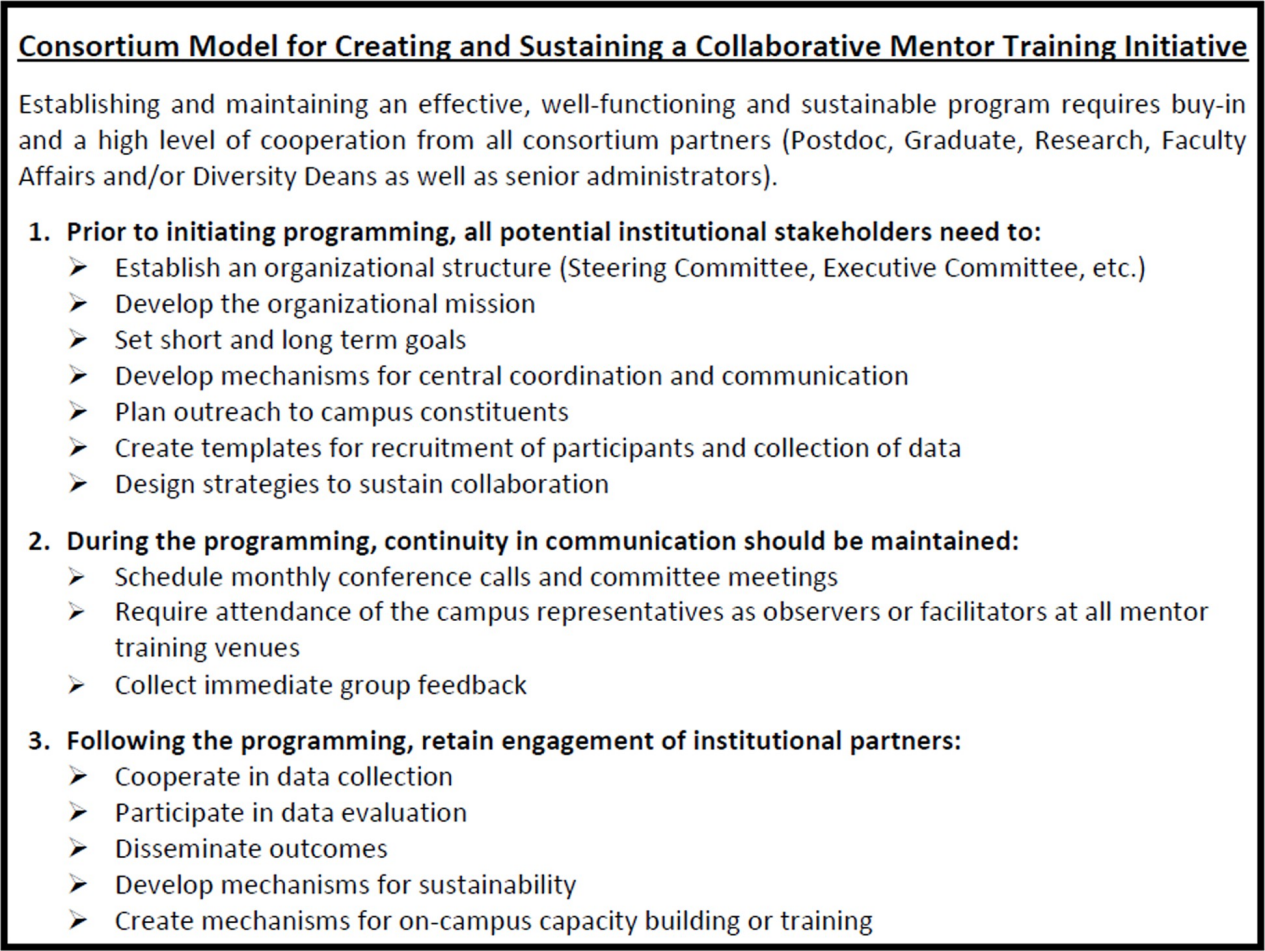
Consortium model for creating and sustaining a collaborative mentor training initiative.

Although most institutions espouse a desire for greater diversity, there remains a disconnect between intent and the structural and systemic reality in higher education [37]. This disconnect is often the crux of why diversity initiatives fail. Increasingly, failure is most evident at the transition from postdoc to faculty. A remedy will require coordinated efforts at both the individual and the institutional levels: such efforts have been the dual goals of the NRMN-CAN initiative. In fact, while evidence suggests that “Mentoring-Up” training for trainees, especially postdocs, and Mentor Facilitator training for faculty contribute to mentee success individually (see references in Introduction), to our knowledge providing both types of interventions to participants at the same institutions, followed by a campus-based “spread” model has not been reported previously. As well, this pilot program has revealed useful strategies that were developed through a consensus process by the Big Ten representatives, which may be replicated by other clusters of universities (Fig 5). These are briefly discussed below and provide the nidus for future studies.

For the NRMN-CAN initiative, we employed the specific curriculum, “Mentoring-Up”, developed for postdocs and PhD students to more effectively guide the mentoring they are receiving, while simultaneously providing the skills for them to become more effective mentors themselves [14]. “Mentoring-Up” training to 102 postdocs was followed by determining whether and how the addition of mentor training designed to nullify social/cognitive barriers, especially for women and UR postdocs, enhanced their career outcomes. Our data show that the majority (70%) of postdocs indicated that mentor training positively impacted their relationship with their Mentors in several important career-enhancing domains (confidence-building, self-efficacy, relationship management, effective negotiation, etc.). As increasingly recognized, mechanisms devoted to learning the skills needed to proactively manage relationships with mentors, which may change over time [38], provide the framework for a lifelong ability to navigate one’s career. Our results indicate that the evidence-based “Mentoring-Up” curriculum has effectively guided the majority of Big Ten Academic Alliance postdoc participants to better understand their mentoring needs, developed strategies to manage their mentoring relationships and empowered them to make critical career decisions, especially to remain in pursuit of an academic career (59%). Our results with the “Mentoring-Up” program are consistent with social cognitive theory [39, 40] in that post-docs and early career scientists self-reported actions such as having more confidence in pursuing an academic career (80%) after participating in the workshops, consistent with increased self-efficacy and -advocacy.

As intended, but not previously demonstrated, mentor training for postdocs significantly (88%) improved their perceived proficiency as mentors, especially for females (75%) and UR (62%) postdocs. Interestingly, postdocs indicated that mentor training also positively influenced their career decisions, especially the confidence to remain in pursuit of an academic career. Although comprehensive data for postdocs are not routinely collected [41], tenure track faculty positions are estimated to represent a small percentage (15%) of postdoc career outcomes [42, 43]. Impressively, 29% of the responding postdocs, predominantly females (75%) and underrepresented postdocs (85%) have successfully migrated to faculty (an additional 3 participants—2 female and 1 UR—who did not respond to this survey also obtained faculty positions, but are not included in our reported data). Some postdocs also indicated that their mentor training and experiences were valuable skills when applying for academic positions and definitely aided in adapting to responsibilities as a faculty mentor. Importantly, 65% of these postdocs stayed within the Big Ten Academic Alliance, i.e., research-intensive institutions. Recruiting underrepresented faculty was one of the desired outcomes for Big Ten Academic Alliance institutional investment in this initiative.

It should be noted that many women and UR individuals leave the academy at the postdoctoral stage, and data suggest that building social identity and connection to discipline can play an important role for retention [31]. This study also suggests that strong mentoring may reduce the significant attrition at this transition stage. Given that almost 30% of underrepresented post-docs who participated in NRMN-CAN “Mentoring-Up” training (Table 5) made a transition to faculty positions, and of this group, 75% were female and 85% were UR, we believe that the NRMN-CAN approach for improved mentor training for postdocs may help address some of the barriers leading to the “leaky pipeline” for women and underrepresented individuals in academia. Additionally, given that the majority of postdocs (92% of the females and 88% of the UR postdocs), reported adopting mentoring skills that they learned from the “Mentoring-Up” training, it would appear that offering mentor training, especially targeting postdocs and junior faculty, may be an important contribution to addressing this unmet need, and ultimately improving the long-term diversity of the academy.

Secondly, the benefits of a multi-institutional, overlapping postdoc and facilitator training session cohort approach cannot be overstated. The NRMN-CAN Program: 1.) marries the benefits of large face-to-face, multi-institutional workshops, which allowed introduction to knowledge experts that subsequently were exchanged between campuses; 2.) leverages unique instructional talent, requiring only modest institutional resources since there was no charge for participants; 3.) provides abundant opportunities for networking to expand the trainee and faculty portfolios of colleagues, especially important for UR participants who may be the lone UR scientist in their home department; 4.) coupled with the coincident group workshops, are the benefits of subsequent Mentor training venues on their home campuses, which provide continuity in process as well as individual peer interactions and additional mentor attention, as twenty (30%) postdocs reported taking advantage of additional mentor training, predominantly (76%) on their home campuses.

This model is generalizable for any group of institutions that individually have small numbers of UR postdocs, limited number of professional development experts, and typically is not able to devote significant resources for career enhancing programming. In the short period (<4 years) of the NRMN-CAN program, the scaling of implementation on home campuses has been significant with 59 facilitators (from training in the first three years) reaching 2,560 graduate students, postdocs, and faculty (ratio ~ 1:40), with an additional 401 in the special campus-award sponsored workshops and 130 in summer programs, for a total of 3,091. Although not included in the numbers reported in this manuscript, we continue to receive reports describing on-going annual mentor training workshops and incorporation of mentor training into a variety of institutional programs. For example, one Facilitator reported i.) integrating mentoring training into their institution’s Presidential Postdoc Fellows program for both the advisors and postdocs; ii.) running a 10 week mentor training course for graduate students and postdocs in Engineering (underscoring the applicability to other disciplines); and iii.) implementing faculty mentor training for four different departments at the request of deans and department chairs (responding to NIH training guidelines). With respect to Mentor training for postdocs, at least four institutions have integrated the full “Mentoring-Up” curriculum annually into their suite of professional development and career-enhancing programming. These reports indicate that the dissemination of mentor training from the NRMN-CAN initiatives is continuing to grow and become established into the institutional training culture. Lastly, while preparing this report, we have been informed that many member institutions have pivoted to remote mentor training venues, suggesting that mentor training can be successfully adopted to virtual implementation for both faculty and trainees.

A unique feature of our approach was to include institutional leadership (Assistant, Associate, and Deans of Diversity, Graduate Studies, Postdoc Offices, Research and/or their senior staff) in annual training events at the Big Ten Conference Center, so that they became completely knowledgeable about our goals, activities, and the administrative support needed for implementing continued training and community-building activities subsequently on their home campuses; i.e., the institutional support needed for providing infrastructure and promoting “buy in” from top leadership to garner resources. Interestingly, faculty reported implementing campus mentor training venues together with administrators (21%) or with other faculty colleagues plus administrators (50%), emphasizing the critical importance of including senior administrators in this capacity-building process; the perceived benefits and institutional impact of these team partnership approaches warrant further investigation. Clearly, offering the facilitator training model for faculty research mentors, as well as Deans and senior administrators, has empowered these institutional participants to collaboratively establish subsequent mentor-training programs on their campuses, as well as sharing expertise among institutions, thus mitigating some of the previously identified barriers to implementation and dissemination of Mentor training [29]. Moreover, building local capacity and fostering sustainability of good mentorship practices is a major step toward institutional transformation. It should be mentioned that all faculty and senior administrators participated as volunteers, without any compensation, in contrast to other mentor-training models. Clearly, all these advantages are generalizable to other academic consortia.

## Conclusion

Promoting institutional transformation and cultural change is one of the most challenging problems in higher education. Clearly, this pilot project has had positive outcomes and has spearheaded development of useful strategies for postdoc career progression and institutional transformation. Importantly, those institutions which participated extensively in sending mentees and mentors and subsequently executing follow-up campus-based activities were those with substantial institutional leadership participation in NRMN-CAN organization and planning. Furthermore, we have presented strong preliminary data on the extent to which the approaches to mentor training have become established institutional practices on member campuses, which is necessary to increase mentor training capacity and long-term sustainability of improved mentoring practices. Thus, there remains a progressive vision among the multi-institutional consortium, based on the longstanding cooperation of the Big Ten Academic Alliance in sharing expertise, leveraging resources, and creating innovative programming, to intra- and inter-institutionally shape meaningful culture change.

## Supporting information

S1 Appendix Postdoctoral mentor training curriculum. Two representative schedules from NRMN-CAN Postdoctoral “Mentoring-Up” workshops are provided. These include a longer and shorter format with the same core competencies covered in both workshops.
(PDF)

S2 Appendix Facilitating mentor training curriculum. A representative schedule from a NRMN-CAN Mentor Facilitator Training workshop is provided.
(PDF)
