# Supplementary material for: Leveraging a collaborative consortium model of mentee/mentor training to foster career progression of underrepresented postdoctoral researchers and promote institutional diversity and inclusion
Source: PLoS One. 2020 Sep 1;15(9):e0238518. doi: 10.1371/journal.pone.0238518 (PMC7462290; doi:10.1371/journal.pone.0238518)
Supplement: S1 Appendix — Two representative schedules from NRMN-CAN Postdoctoral “Mentoring-Up” workshops are provided. These include a longer and shorter format with the same core competencies covered in both workshops. (PDF) [file pone.0238518.s001.pdf]

S1 Appendix. Postdoctoral Mentor Training Curriculum.

NRMN
National Research Mentoring Network

B1G
ACADEMIC ALLIANCE

NRMN-CAN Postdoctoral Mentor Training Agenda

Sunday, March 17, 2019
Aloft Hotel
Conference Area past Main Lobby

5:00pm – 6:00pm	Registration and Networking Reception
6:00pm – 6:30pm	Welcome and Introductions
6:30pm – 7:45pm	Promoting Self-Efficacy
7:45pm – 8:45pm	Work-Life Integration

Monday, March 18, 2019
Big Ten Conference Center, Second Floor, Room 202

7:30am – 8:30am	Breakfast and Networking
8:30am – 10:00am	Maintaining Effective Communication
10:00am – 10:15am	Break
10:15am – 11:15am	Addressing Equity and Inclusion
11:15am – 12:00pm	Lunch
12:00pm – 1:45pm	Aligning Expectations and Cultivating Ethical Behavior
1:45pm – 2:00pm	Break
2:00pm – 3:30pm	Fostering Independence and Promoting Professional Development
3:30pm –4:00pm	Articulating Mentoring Philosophy and Next Steps

NRMN-CAN Postdoctoral Mentor Training Workshop Agenda
Big Ten Conference Center, Rosemont, IL
June 19-21, 2016

Sunday, June 19, 2016 - Arrival and Networking at Aloft Chicago O'Hare Hotel

Time	Activity	Location
7:00-8:00pm	Evening Networking Reception	Aloft Hotel Lobby and XYZ Bar

Monday, June 20, 2016 - Big Ten Conference Center

7:30-8:30am	Breakfast, Registration and Networking	2nd Floor Lobby
8:30-9:00am	Welcome and Introductions	Room 205
9:00-10:30am	Maintaining Effective Communication	Room 205
10:30-10:45am	*BREAK*	
10:45-12:00pm	Addressing Equity and Inclusion	Room 205
12:00-1:45pm	*Keynote Address*<br>"Mentoring and Leading From Within: Who Knows YOU Best?"<br>Dr. Jabbar Bennett, Northwestern University<br>*Lunch available in lobby*	Room 205
1:45-2:45pm	Aligning Expectations	Room 205
2:45-3:45pm	Promoting Research Self-Efficacy	Room 205
3:45-4:00pm	*BREAK*	
4:00-4:45pm	Fostering Independence	Room 205
4:45-5:30pm	Cultivating Ethical Behavior	Room 205
5:30-6:30pm	Networking Reception	Dining Area

NRMN-CAN Postdoctoral Mentor Training Workshop Agenda
Big Ten Conference Center, Rosemont, IL
June 19-21, 2016

Tuesday, June 21, 2016 - Big Ten Conference Center

Time	Activity	Location
7:30am	Breakfast	2nd Floor Lobby
8:00-9:00am	Promoting Professional Development	Room 205
9:00-9:10am	*BREAK*	
9:10-11:00am	Enhancing Work-Life Integration	Room 205
11:00-11:05am	*BREAK*	
11:05-12:00pm	Articulating your mentoring philosophy and next steps	Room 205
12:00pm	*Boxed lunches to go*	
