# Supplementary material for: Leveraging a collaborative consortium model of mentee/mentor training to foster career progression of underrepresented postdoctoral researchers and promote institutional diversity and inclusion
Source: PLoS One. 2020 Sep 1;15(9):e0238518. doi: 10.1371/journal.pone.0238518 (PMC7462290; doi:10.1371/journal.pone.0238518)
Supplement: S2 Appendix — A representative schedule from a NRMN-CAN Mentor Facilitator Training workshop is provided. (PDF) [file pone.0238518.s002.pdf]

S2 Appendix. Facilitating Mentor Training Curriculum.

NRMN-CAN Facilitating Mentor Training Workshop
Monday, March 18 – Tuesday, March 19, 2019
Big Ten Conference Center, Rosemont, IL

Day 1: Experiencing Research Mentor Training

Time	Activity
7:30 - 8:30	Registration, Networking and Breakfast
8:30 - 9:30	Introduction to Research Mentor Training (Room 205)
9:30 - 10:30	Competency 1: Maintaining Effective Communication
10:30 - 10:45	Break
10:45 - 11:45	Competency 2: Aligning Expectations
11:45 - 12:00	Introduction to Practice Facilitation
12:00 - 1:00	Lunch
1:00 - 1:45	Competency 3: Fostering Independence
1:45 - 2:30	Competency 4: Promoting Professional Development
2:30 - 2:45	Break
2:45 - 3:45	Competency 5: Addressing Equity and Inclusion
3:45 - 3:50	Mentoring Reflection
3:50 - 4:30	Next Steps: Prepare for Tomorrow's Practice Facilitation Session
4:30 - 6:30	Break
6:30 - 7:30	Networking Dinner Reception in Aloft Hotel Exchange conference room (past the bar in the lobby)

S2 Appendix. Facilitating Mentor Training Curriculum.

NRMN-CAN Facilitating Mentor Training Workshop

Monday, March 18 – Tuesday, March 19, 2019

Big Ten Conference Center, Rosemont, IL

Day 2: Facilitating Research Mentor Training

Time	Activity
7:30 - 8:30	Breakfast and Networking
8:30 - 9:45	Introduction to Facilitator Training
9:45 - 10:00	Break and Transition to Breakout Rooms (Rooms 202, 206, 214, 215, 246)
10:00 - 12:00	Round 1 (10:00 - 10:30); Round 2 (10:30 - 11:00); Round 3 (11:00 - 11:30); Round 4 (11:30 - 12:00)
12:00 - 1:00	Lunch
1:00 - 1:30	Large Group Debrief
1:30 - 2:45	Implementation Resources
2:45 - 3:00	Break
3:00 - 4:00	Implementation Planning
4:00 - 4:30	Wrap Up and Next Steps
